# Long non-coding RNA rhabdomyosarcoma 2-associated transcript contributes to neuropathic pain by recruiting HuR to stabilize DNA methyltransferase 3 alpha mRNA expression in dorsal root ganglion neuron

**DOI:** 10.3389/fnmol.2022.1027063

**Published:** 2023-02-22

**Authors:** Xinying Guo, Gaolong Zhang, Weihua Cai, Fa Huang, Jingwen Qin, Xingrong Song

**Affiliations:** ^1^Department of Anesthesiology, Guangzhou Women and Children’s Medical Center, Guangzhou Medical University, Guangdong Provincial Clinical Research Center for Child Health, Guangzhou, China; ^2^Department of Anesthesia, McGill University, Montreal, QC, Canada

**Keywords:** neuropathic pain, dorsal root ganglia, *Rmst* lncRNA, HuR, DNA methyltransferase 3 alpha (*DNMT3A*), mRNA stability

## Abstract

**Introduction:**

Long non-coding RNAs (lncRNAs) act as key regulators in multiple human diseases. In particular, the dysfunction of lncRNAs in dorsal root ganglion (DRG) contributes to the pathogenesis of neuropathic pain (NP). Nevertheless, the role and mechanism of most lncRNAs in NP remain unclear.

**Methods:**

Two classic chronic NP models, including L4 spinal nerve ligation (SNL) model and chronic constriction injury (CCI) of the sciatic nerve, were performed. Mechanical allodynia and heat hyperalgesia were used to evaluate neuropathic pain. DRG microinjection was used to deliver agents into DRG. qRT-PCR, immunofluorescence, immunoprecipitation, western blotting, siRNA transfection, AAV transduction were performed to investigate the phenotypes and molecular basis.

**Results:**

Here, we discovered that *Rmst* as a lncRNA was specifically expressed in *Atf3*^+^ injured DRG neurons and significantly upregulated following peripheral nerve damage. *Rmst* overexpression by direct DRG injection of AAV5-*Rmst* causes neuropathic symptoms in the absence of nerve damage. Conversely, blocking *Rmst* expression in injured DRGs alleviated nerve injury-induced pain hypersensitivities and downregulated *Dnmt3a* expression. Furthermore, we found peripheral nerve damage induced *Rmst* increase could interact with RNA-binding protein HuR to stabilize the *Dnmt3a* mRNA.

**Conclusion:**

Our findings reveal a crucial role of *Rmst* in damaged DRG neurons under NP condition and provide a novel target for drug development against NP.

## Introduction

Chronic pain has been a perplexing problem for decades. Globally, approximately one-fifth of people suffer from chronic pain (Tompkins et al., 2017), and about thirty percent of these patients have gone through the symptoms of neuropathic pain (NP) (van Hecke et al., 2014). Patients with NP have detrimental effects on their quality of life and ability. In addition to personal suffering, chronic pain is a substantial financial burden on society, and lies billions of dollars every year (van Velzen et al., 2020). However, there are limited treatments available for NP. Dorsal root ganglia (DRG) neuron is responsible for conveying the nociception in peripheral nerve injury. Peripheral nerve damage-induced maladaptive alterations at transcriptional and translational levels of pain-associated genes in the primary afferents of DRG neurons contribute to these abnormal spontaneous activities and subclinical behavioral patterns (Li et al., 2020; Pan et al., 2021). Hence, identifying new targets and mechanisms in damaged DRGs could open a novel avenue against NP.

As potent and multifaceted roles of long non-coding RNAs (lncRNAs) in gene regulation, lncRNAs are paid much attention in many human illnesses, including NP (Wu et al., 2016, 2019). However, the function of most lncRNAs in NP is not thoroughly understood. A lncRNA called rhabdomyosarcoma 2-associated transcript (*Rmst*) was essential for neuronal differentiation (Ng et al., 2012) and neurogenesis (Ng et al., 2013). Although previous studies showed that *Rmst* was involved in neurological disorders (Hou and Cheng, 2018; Ma et al., 2021; Zhao et al., 2021; Li et al., 2022), little is known about its role in NP.

DNA methyltransferase 3 alpha gene encodes the *Dnmt3a* protein and is responsible for catalyzing 5-methylcytosine methylation. The aberrant regulation of Dnmt3a is implicated in multiple nervous diseases (Feng et al., 2005; Clemens and Gabel, 2020), especially in NP (Guo et al., 2019). Increased Dnmt3a have been found in injured DRG (Shao et al., 2017; Zhao et al., 2017). What’s more, the increased-Dnmt3a was able to mediate the epigenetic inaction of the voltage-dependent potassium channel subunit (Kcna2) *via* DNA hypermethylation of Kcna2 promoter region (Zhao et al., 2017). Depletion of Dnmt3a in the injured DRGs effectively attenuated NP by restoring *Kcna2* expression (Zhao et al., 2017, p. 3). These studies suggested that Dnmt3a plays a pivotal role in regulating DNA methylation of nerve damage related gene alterations. Nevertheless, it is unclear how peripheral nerve damage triggers the activation of Dnmt3a in injured DRG.

Here, we sought to characterize a lncRNA rhabdomyosarcoma 2-associated transcript (*Rmst*) in injured DRG neurons was substantially increased in NP. The nerve damage triggered *Rmst* expression in injured DRGs contributes to regulating Dnmt3a through interaction with RNA binding protein HuR in NP. Thus, the role and mechanism of *Rmst* may provide novel and insightful directions for NP management in clinic.

## Materials and methods

### Bioinformatics

RNA sequencing dataset for mouse DRG after peripheral nerve injury was obtained from previous research (Wu et al., 2016). The mouse DRG was harvested 7 days after L4 spinal nerve ligation (SNL) model and sequencing was performed on the Illumina HiSeq2500 platform with 2 × 100-bp paired-end reads.

For scRNA-seq dataset for mouse DRG after peripheral nerve injury, we obtained the cell count matrix and metadata from Gene Expression Omnibus (GEO) with the series record GSE155622 (Wang K. et al., 2021). The mouse DRG was harvested for Smart-seq2 as smart-seq2 was better to captured low abundance transcripts as well as more lncRNAs (Wang X. et al., 2021). All metadata was also obtained from GSE155622.

### Animals

C57BL/6J adult mice were from SPF Biotechnology Co., Ltd (Beijing, China). A 12-h light-dark cycle environment with unlimited access to food and water was used to house mice. All processes were endorsed by the Animal Care and Use Committee at Guangzhou Medical University.

### Chronic neuropathic pain model

Two classic chronic NP models, including L4 SNL model and chronic constriction injury (CCI) of the sciatic nerve, were performed as described previously (Li et al., 2020). SNL model was established by tightly ligating L4 spinal nerve distal to DRG with 7-0 silk suture. L4 spinal nerve was exposed in the matched sham mice, however, the L4 spinal nerve was neither ligated nor transected. CCI model was established by loosely ligated the sciatic nerve at three spots with 1 mm-intervals by 7-0 silk suture. Sham animals did not receive the ligation of the sciatic nerve.

### Behavioral tests

As previous described, mechanical allodynia was quantified by measuring paw withdrawal frequency by low (0.07 g) and median (0.4 g) von Frey filaments (Stoelting Co., Wood Dale, IL, USA) (Li et al., 2020). We use two calibrated plastic filaments to stimuli the central of plantar surface of hind paws. A positive response is quick withdrawal of the paw. Mice were totally received 10 applications. The paw withdrawal frequency describes the positive withdrawal responses within 10 applications.

Heat hyperalgesia was quantified by measuring paw withdrawal latencies after heat stimulation as described (Li et al., 2020). All mice before behavior test were left in a glass surface in individual plexiglas cages. A beam of light was applied to the central of hind paw. The performance of a positive response is a swift raise of the hind paw. The Model 336 Analgesia Meter (IITC Inc., Life Science Instruments. Woodland Hills, CA, USA) was automatically records the withdrawal latency from heating source. 4–5 trials on each side were performed at intervals of 5 min.

### Dorsal root ganglion microinjection

As described previously, DRG microinjection was carried out (Li et al., 2020). After 3-cm-long skin incision, we firstly exposed the corresponding spinal nerve (L4 and/or L3). After that, we used rongeur to remove the unilateral articular processes for DRG microinjection. The glass micropipette was carefully inserted into the exposed ipsilateral L4 and/or L3 DRGs and 1 μl of either siRNA solution or viral solution was injected into L4 and/or L3 DRG. The injection was performed at a rate of 10 nl/s. After the injection was completed, the pipette was left in place for 10 min before removal to allow the fluid to distribute and the pressure within the DRG to equalize. The skin was sutured with 6-0 silk and mice were kept on a heating pad. All reagent and surgical instruments are sterilized in advance.

### Dorsal root ganglion neuronal culture

We performed DRG neuron cultures as described (Li et al., 2020). We first prepared complete neurobasal medium (CNM) including 10% fetal bovine serum, and 1x antibiotics, 2% B-27 supplement, and 1% GluMax supplement. 3–4 weeks mice were used for collecting DRGs. The collected DRGs were incubated with collagenase solution including dispase, collagenase type I in HBSS. All reagents are from Thermofisher Scientific Company (Waltham, MA, USA).

### Quantitative real-time RT–PCR

The TRIzol Reagent (Cat. No:15596026, Invitrogen Corporation, Carlsbad, CA, USA) was used for extracting RNA from DRGs, followed by reverse transcription using PrimeScript RT Master Mix (Cat. No: RR036A, Takara Bio Inc, Shiga, Japan). Quantitative PCR were performed using TB Green Premix Ex Taq II (Tli RNaseH Plus) (Cat. No: RR820A, Takara Bio Inc, Shiga, Japan) on a CFX96 Touch Real-Time PCR Detection System (Bio-Rad Laboratories, Inc., Hercules, CA, USA). Finally, relative fold changes of each gene were calculated by ΔCt method (2^–ΔΔ*Ct*^). Supplementary Table 1 included all primer information.

### Ribonucleic acid stability assay

Primary neurons grew in 6-well plate and were treated with actinomycin D (Cat. No:A1410, Sigma-Aldrich, Burlington, MA, USA) for testing the mRNA stability. After that, we collected neurons at 0, 2, 4, 8, and 12 h after actinomycin D treatment for RNA extraction.

### Nuclear/cytoplasmic ribonucleic acid fraction isolation

Cytoplasmic and Nuclear RNA purification Kit were purchased from Norgen Biteck Corp. (Cat. No: NGB-21000, Thorold, ON, Canada). After Nuclear and cytoplasmic RNA fraction isolation, various gene expression levels in both nuclear and cytoplasmic fractions of all samples were calculated by RT-PCR as protocol above.

### Nuclear/cytoplasmic protein isolation

Nuclear and cytoplasmic protein were separated using the NE-PER nuclear and cytoplasmic extraction reagents (Cat. No: 78833, Thermofisher Scientific Company, Waltham, MA, USA) following the manufacturer’s instructions. The collected protein was aliquoted and stored at −80°C.

### Plasmids constructs and virus production

The pAAV-CMV-mRmst-:WPRE vector (vector ID: VB220510-1032tdc) and AAV5-Rmst virus packaging was designed and constructed to overexpress the Rmst expression by VectorBuilder Company (Chicago, IL, USA). Briefly, the full-length cDNA of Rmst (NR_028262.1) was amplified by RT-PCR. After that, double enzyme-digested PCR products were ligated into the mammalian ncRNA expression AAV vector as a plasmid. AAV5-Gfp (vector ID: VB150925-10026) is used as negative control. SiRNA for *Rmst* and *Dnmt3a* were designed and produced by Tsingke Biotechnology Co., Ltd. (Beijing, China). All siRNA sequencing used in this work were listed at Supplementary Table 1.

### Immunohistochemistry staining

Mice were perfused with 4% PFA after deep isoflurane before being analyzed by immunohistochemistry. The DRGs were collected and post-fixed in 4% PFA overnight, followed by dehydrating in 30% sucrose for two nights at 4°C. Finally, the DRGs were sectioned at 15–20 μm and kept them in −80°C refrigerator.

Before primary antibody incubation, the section was blocked in 1X PBS with 10% donkey serum and 0.3% Triton X-100. The sections were then incubated with anti-DNMT3a (Santa Cruz, Dallas, TX, USA) overnight at 4°C followed by incubating secondary antibody conjugated to Cy3 (1:500, Jackson ImmunoResearch, West Grove, PA, USA) for 2 h. Finally, the sections were mounted using Fluoroshield™ with DAPI (Cat. No: F6057, Sigma-Aldrich, Burlington, MA, USA).

### Ribonucleic acid-binding protein immunoprecipitation (RIP)

The Magna RIP Kit were purchased from EMD Millipore (Burlington, MA, USA) company for RIP assay. A anti-HuR antibody (Santa Cruz, Dallas, TX, USA) was used in RIP assay. After purification of RNA, RT-PCR was performed following the previous protocol.

### Western blotting

The collected protein was firstly separated using SDS-PAGE electrophoresis on the basis of size, followed by moving to PVDF membranes with appropriate size. The blot was then immediately placed in 5% fresh non-fat milk powder for blocking for 1 h. Next, the appropriate primary and secondary antibodies were used to incubate the transferred membrane according to the recommended dilution and time in datasheet. The rabbit anti-DNMT3b (1:500), rabbit anti-DNMT3a (1:500), and rabbit anti-histone H3 (1:1,000) were purchased from Cell Signaling Technology (Danvers, MA, USA). The mouse anti-HuR (1:500) and rabbit anti-GAPDH (1:1,000) were purchased from Santa Cruz company (Dallas, TX, USA). Membranes were visualized by the Clarity Western ECL Substrate (Cat. No: 170-5060, Bio-Rad Laboratories, Inc., Hercules, CA, USA), exposed by ChemiDoc Touch (Bio-Rad Laboratories, Inc., Hercules, CA, USA) and analyzed by Image J.

### Statistical analysis

Sample size estimation was based on certain assumptions, including significance level, expected placebo mean, expected treatment mean, standard deviation, power, expected dropout. In our case, expected placebo mean, expected treatment mean, standard deviation, and expected dropout were depended on our pilot studies and previous report in the field (Li et al., 2020; Pan et al., 2021). After that, sample sizes were calculated using nQUERY software, assuming a significance level of 0.05, 90% power and homogeneous variances for the 2 samples to be compared, with the means and SEM for different parameters predicted from pilot study. The data is presented as mean + SEM and analyzed by GraphPad Prism 8. The Shapiro–Wilk test was used for normal distribution test. If the data passed the normality test, the *t*-test or ANOVA was used in this study by GraphPad Prism 8 software according to experimental design and the detailed statistical method was listed in figure legend. The interaction of factors after ANOVA is provided in result. A *P*-value of less than 0.05 was considered statistical significant.

## Results

### Increased rhabdomyosarcoma 2-associated transcript in damaged dorsal root ganglions was found in neuropathic pain

**FIGURE 1:**
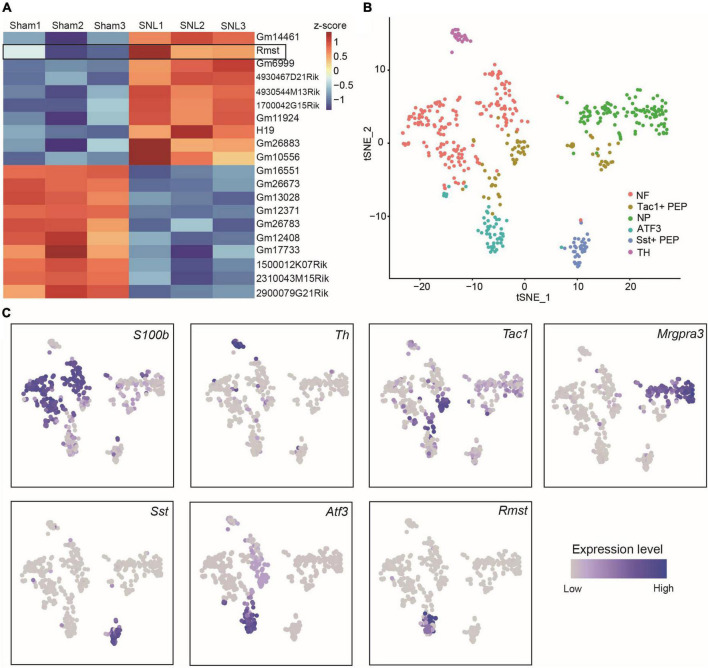
Rhabdomyosarcoma 2-associated transcript (*Rmst*) was specifically expressed in *Atf3*^+^ injured neurons. **(A)** The 10 most up- and downregulated lncRNAs in L4 DRGs after SNL model. The heatmap was created using Z-score values obtained from RNA-seq dataset. **(B)** TSNE plot of all cells from DRGs after nerve injury. Each dot was color-coded and annotated by neural subtypes. NF: neurofilament, NP: non-peptidergic neurons, TH*:* tyrosine hydroxylase, PEP: peptidergic neurons. **(C)** TSNE plots showing scRNA-seq data, colored by gene expression level, showing *S100b, Th, Tac1, Mrgpra3, Sst, Atf3*, and *Rmst* expression.

As the critical role of lncRNAs during the formation of NP, we extracted the 10 most up-and downregulated lncRNAs in L4 DRGs 7 days after SNL model (Figure 1A) from the previous RNA sequencing dataset (Wu et al., 2016). Among them, we found *Rmst*, a previously reported brain specific lncRNA (Ng et al., 2012), was also expressed in damaged DRGs (Figure 1A). As previous research has been found that *Rmst* was specific to neuron (Briese et al., 2016), we are wondering the distribution of *Rmst* in the neuron subtype after nerve injury. Thus, we analyzed the scRNA sequencing dataset from DRGs with peripheral nerve damage (Wang K. et al., 2021). As the smart-seq2 technology was better to captured low abundance transcripts as well as more lncRNAs (Wang X. et al., 2021), we focused on the smart-seq2 analysis to explore DRG neuron subtype. In t-distributed stochastic neighbor embedding (tSNE), 6 traditional classification neuronal subtype was found (Figure 1B), including *S100b*^+^ neurofilament (NF), *Mgpra3*^+^ non-peptidergic neurons (NP), *Th*^+^ tyrosine hydroxylase (TH), *Tac1*^+^ and *Sst*^+^ peptidergic neurons (PEP), and *Atf3*^+^ injured neuron (Figures 1B,C). Interestingly, we found *Rmst* was specifically expressed in *Atf3*^+^ injured neuron (Figure 1C), suggesting that *Rmst* may be involved in NP.

**FIGURE 2:**
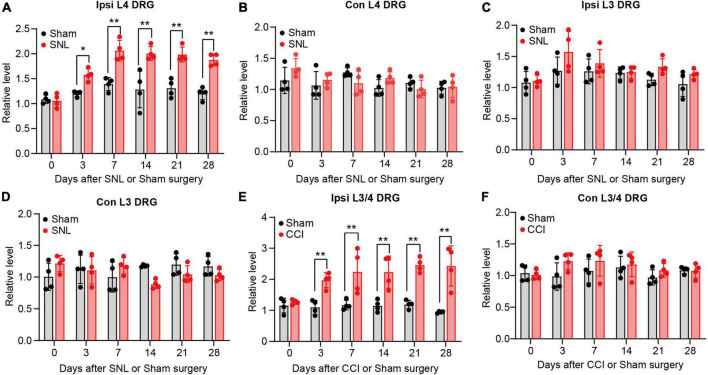
Rhabdomyosarcoma 2-associated transcript (*Rmst*) upregulation was found in damaged DRG of mice after SNL or CCI model. **(A–D)** Level of *Rmst* in ipsilateral L4 DRGs **(A)**, contralateral L4 DRGs **(B)**, ipsilateral L3 DRGs **(C)**, and contralateral L3 DRGs **(D)** after SNL or sham surgery *n* = 16. **(E,F)** Levels of *Rmst* in the ipsilateral **(E)** and contralateral **(F)** side L3/4 DRGs after CCI or sham surgery *n* = 8. Two-way ANOVA followed by *post-hoc* Tuckey test. *P < 0.05 and **P < 0.01 compared with sham group at each time point.

To provide the adequate evidence, we performed animal neuropathic pain model to validate the result. We found lncRNA *Rmst* expression was increased early and persistently at least 28 days in damaged DRGs after SNL surgery but not sham surgery (Figure 2A) [*F*(5,36) = 5.942]. To be specific, *Rmst* expression was elevated 1.58-fold on day 3, 2.07-fold on day 7, 2.01-fold on day 14, 1.99-fold on day 21, 1.88-fold on day 28 after SNL but not in the contralateral L4 DRGs and L3 DRGs (Figures 2A–D) [Figure 2B: *F*(5,36) = 1.896; Figure 2C: *F*(5,36) = 0.7694; Figure 2D: *F*(5,36) = 2.980]. A similar phenomenon was observed in another NP mouse model called CCI model (Figures 2E,F) [Figure 2E: *F*(5,36) = 3.530]; [Figure 2F: *F*(5,36) = 0.8966]. The *Rmst* expression in damaged DRGs were elevated 1.97-fold on day 3, 2.23-fold on day 7, 2.23-fold on day 14, 2.46-fold on day 21, 2.3-fold on day 28 after CCI model (Figure 2E). Taken together, our data showed peripheral nerve damage could trigger the elevated *Rmst* expression in the damaged DRGs that maintained at least until 1-month post-SNL, suggesting that *Rmst* may take part in the nerve damage-induced NP.

### Blocking rhabdomyosarcoma 2-associated transcript expression in damaged dorsal root ganglions alleviated neuropathic pain

**FIGURE 3:**
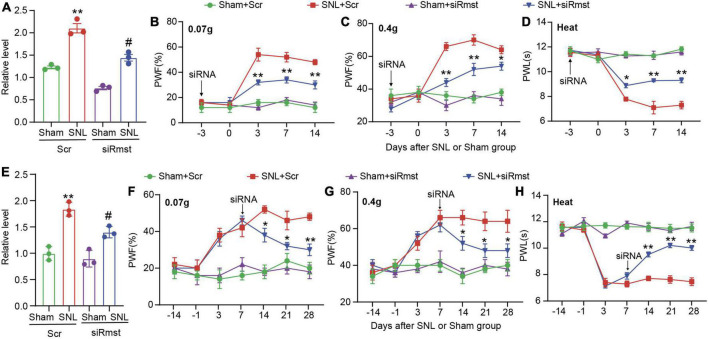
Blocking nerve damage triggered rhabdomyosarcoma 2-associated transcript (*Rmst*) expression in DRG mitigated the nerve damaged-induced nociceptive hypersensitivity. **(A)** Level of *Rmst* in the L4 DRGs on day 14 after nerve injury model in presence of siRmst or Scr. *n* = 12 mice, One-way ANOVA followed by *post-hoc* Tukey test, **P < 0.01 compared with Sham + Scr group and ^#^*P* < 0.05 compared with SNL + Scr group. **(B–D)** The PWF to von Frey filament **(B,C)** and the PWL to thermal stimuli **(D)** on the development period of NP. *n* = 5 mice, two-way ANOVA followed by *post-hoc* Tukey test, *P < 0.05 and **P < 0.01 compared with the SNL + Scr group. **(E)** Level of *Rmst* in the L4 DRGs on day 28 after nerve injury model in presence of siRmst or Scr. *n* = 12 mice, One-way ANOVA followed by *post-hoc* Tukey test, **P < 0.01 compared with Sham + Scr group and ^#^*P* < 0.05 compared with SNL + Scr group. **(F–H)** The PWF to von Frey filament **(F,G)** and the PWL to thermal stimuli **(H)** on the maintenance period of NP. *n* = 5 mice, two-way ANOVA followed by *post-hoc* Tukey test, *P < 0.05 and **P < 0.01 compared with SNL + Scr group. Paw withdrawal frequency: PWF; Paw withdrawal latency: PWL.

As the apparent change of *Rmst* in damaged DRGs, we further ask whether blocking *Rmst* expression in injured DRG could alleviate pain hypersensitivity. We first confirmed that DRG microinjection of siRmst, but not Scr, 3 days before SNL could block SNL-induced increased *Rmst* expression (Figure 3A) [*F*(3,8) = 65.29]. Microinjection of siRmst also slightly reduced basal expression of *Rmst* expression (Figure 3A). More importantly, we found pre-microinjection of siRmst could ameliorate SNL-induced nociceptive hypersensitivities, including mechanical allodynia (Figures 3B,C) [Figure 3B: *F*(12,80) = 9.633; Figure 3C: *F*(12,80) = 10.15] and heat hyperalgesia (Figure 3D) [Figure 3D: *F*(12,80) = 17.99]. Neither Scr nor siRmst changed basal paw response to mechanical or heat stimuli while mice received sham surgery (Figures 3B–D). We then ask the role of *Rmst* during the maintenance period after SNL model. We first confirmed that DRG microinjection of siRmst in maintenance period after SNL (Figure 3E) [*F*(3,8) = 30.85]. As expected, siRmst delivery through DRG microinjection on day 7 post-SNL rescued pain hypersensitivity (Figures 3F–H) [Figure 3F: *F*(18,112) = 3.614; Figure 3G: *F*(18,112) = 3.312; Figure 3H: *F*(18,112) = 20.60]. Our data strongly support that nerve damage triggered nociceptive hypersensitivity may be attributed to elevated *Rmst* expression in the damaged DRG.

### Dorsal root ganglion long non-coding RNA *Rmst* overexpression produces nociceptive hypersensitivity

**FIGURE 4:**
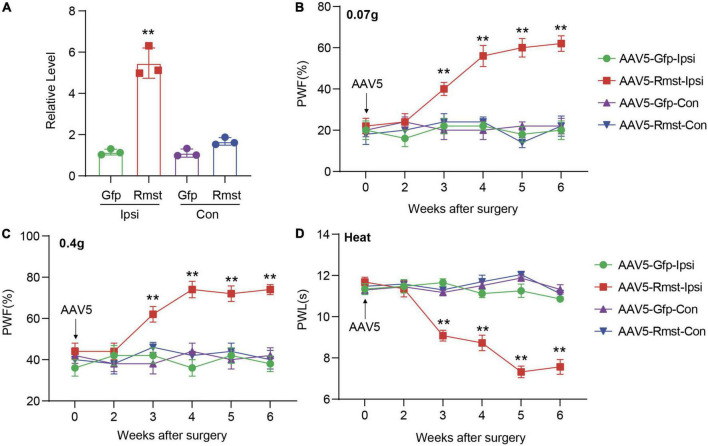
Overexpression of DRG rhabdomyosarcoma 2-associated transcript (*Rmst*) produced nociceptive hypersensitivity in naïve mice. **(A)** *Rmst* expression in L3/4 DRGs 6 weeks after receiving AAV5-Rmst or AAV5-Gfp. *n* = 6 mice, two-tailed unpaired Student’s *t-*test, **P < 0.01 compared with AAV5-Gfp group. **(B–D)** The PWF to von Frey filament **(B,C)** and the PWL to thermal stimuli **(D)** on mice with DRG microinjection of AAV5-Rmst or AAV5-Gfp. *n* = 6, two-way ANOVA followed by *post-hoc* Tukey test, **P < 0.01 compared with AAV5-Gfp-Ipsi group.

Next, we asked if DRG *Rmst* overexpression in neuron is sufficient for NP production. As the previous paper has reported *Rmst* was specifically expressed in the neuron (Briese et al., 2016), we utilized Adeno-associated Virus 5 (AAV5) following the previous papers (Li et al., 2020) to package *Rmst* full-length vector (AAV5-Rmst) and microinjected to the ipsilateral DRG to overexpress the *Rmst* expression. As a proof of concept, we found DRG microinjection of AAV5-Rmst in the ipsilateral side could particularly increase *Rmst* expression in ipsilateral side but neither contralateral side nor AAV5-Gfp (Figure 4A) [*F*(3,8) = 84.17]. More importantly, pain symptoms (Figures 4B–D) [Figure 4B: *F*(15,96) = 5.679; Figure 4C: *F*(15,96) = 3.432; Figure 4D: *F*(15,96) = 14.98] were induced third weeks after *Rmst* overexpression in DRG of naïve mice. It means that, even without nerve damage, DRG *Rmst* overexpression in neuron could result in NP-like symptoms.

### Rhabdomyosarcoma 2-associated transcript participated in the nerve damage induced dorsal root ganglion DNA methyltransferase 3 alpha expression after spinal nerve ligation

**FIGURE 5:**
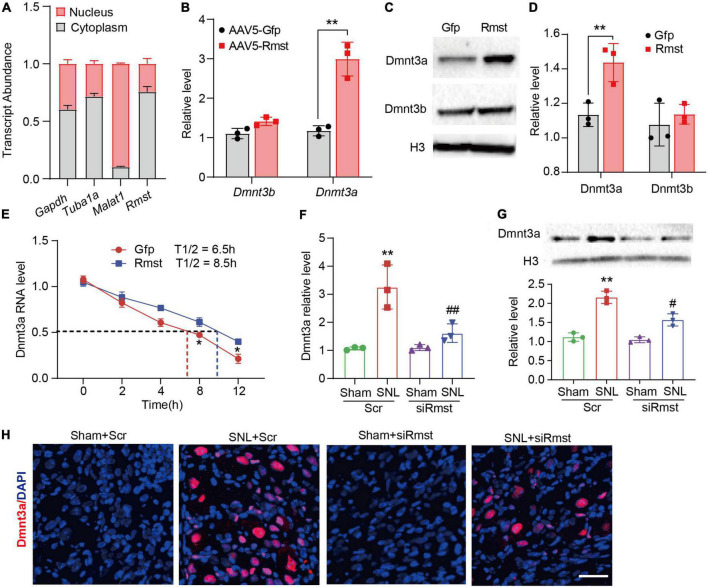
Dorsal root ganglion (DRG) rhabdomyosarcoma 2-associated transcript (*Rmst*) participated in the nerve damage induced Dnmt3a expression by stabilizing *Dnmt3a* mRNA. **(A)** Transcript abundance of cytoplasmic and nuclear RNA fractions for *Gapdh* mRNA, *Tuba1a* mRNA, *Malat1* mRNA, and *Rmst* mRNA from mouse DRGs. *n* = 3 mice. **(B)** Level of *Dnmt3a* and *Dnmt3b* mRNA expression in DRG neuron with AAV5-Rmst. *T*-test was used for statistic analysis. **P < 0.01 versus AAV5-Gfp group. **(C,D)** Dnmt3a and Dnmt3b protein expression in DRG neuron with AAV5-Rmst. *T*-test was used for statistic analysis. **P < 0.01 versus AAV5-Gfp group. **(E)** Primary DRG neurons with the treatment of ActD (5 μg/mL) at multiple time point as shown were treated with AAV5-Rmst or AAV5-Gfp. *n* = 3 biological repeats. *P < 0.05 versus AAV5-Rmst group. **(F,G)** Levels of *Dnmt3a* mRNA **(F)** and protein expression **(G)** in ipsilateral L4 DRGs of SNL mice pre-received with siRmst or Scr. *n* = 12, one-way ANOVA followed by *post-hoc* Tukey test, **P < 0.01 versus Sham + Scr group and ^#^*P* < 0.05, ^##^*P* < 0.01 versus SNL + Scr group. **(H)** Immunostaining for the ipsilateral L4 DRGs showed Dnmt3a protein expression in mice post-SNL and pre-delivered with siRmst or Scr. Scale bar = 40 μm.

Next, the detailed mechanism of *Rmst* involved in NP was investigated. As the subcellular location of lncRNA can provide significant information on its function, we utilized cytoplasmic and nucleus RNA extraction protocol to purify populations of subcellar RNA fractions. Consistent with previously report *in vitro* (Zhao et al., 2021), *Rmst* in naïve mouse DRG was distributed predominantly in the cytoplasm (Figure 5A). Cytoplasmic lncRNAs can function in the posttranscriptional gene expression through mRNA stability and translation (Rashid et al., 2016). It has been reported that *Rmst* could upregulate DNA methyltransferase 3 (Dnmt3) by increasing the stability for its mRNA (Peng et al., 2020) in MCF7 cells, a breast cancer related epithelial cell line. We then examined whether overexpression of *Rmst* could also increase the *Dnmt3a* and *Dnmt3b* mRNA in primary DRG neuron. Surprisingly, only *Dnmt3a* mRNA as well as protein level increased but not Dnmt3b was observed in cultured neurons co-transduced with AAV5-Rmst (Figures 5B–D). In fact, Dnmt3a in primary afferent neurons was reported to participate in NP by repressing the potassium voltage-gated channel Kv1.2 encoded by *Kcna2* (Zhao et al., 2017). To elucidate whether *Rmst* participated in regulating *Dnmt3a* mRNA stability, actinomycin D was used to inhibit the RNA synthesis. We found that *Rmst* could stabilize *Dnmt3a* mRNA transcripts (Figure 5E). The half-life of *Dnmt3a* mRNA was around 8.5 h for *Rmst* overexpressed neurons, as compared to 6.5 h for Gfp control (Figure 5E) [*F*(4,20) = 2.384]. Next, we found microinjection of siRmst, but not Scr, could abolish the SNL- induced Dnmt3a increases (Figure 5F) [*F*(3,8) = 17.11]. Decreased Dnmt3a protein in damaged DRG with the siRmst microinjection after SNL was observed in the nucleus (Figures 5G,H) [*F*(3,8) = 45.02].

**FIGURE 6:**
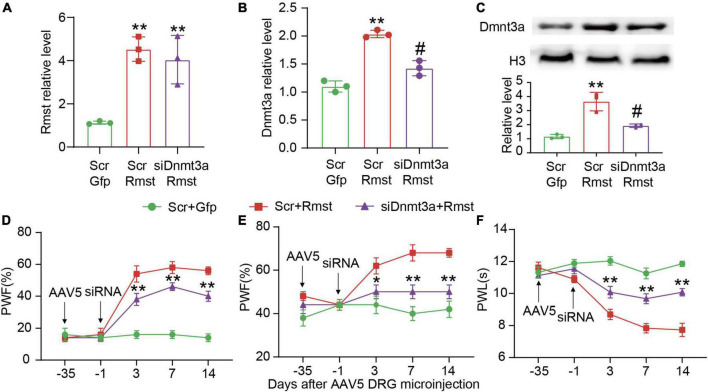
Blocking DNA methyltransferase 3 alpha (Dnmt3a) expression mitigated the rhabdomyosarcoma 2-associated transcript (*Rmst*) triggered-mechanical allodynia and heat hyperalgesia. **(A–C)** *Rmst* mRNA **(A)**, *Dnmt3a* mRNA **(B)**, and Dnmt3a protein **(C)** in mice after microinjection with siDnmt3a (Scr as control) and AAV5-Rmst (AAV5-Gfp as control). **P < 0.01 versus AAV5-Gfp + Scr group and ^#^*P* < 0.05 versus AAV5-Rmst + Scr group. **(D–F)** The ipsilateral PWF to von Frey filament **(D,E)** and the PWL to thermal stimuli **(F)** after microinjection of siDnmt3a or Scr in mice pre-received with AAV5-Rmst or AAV5-Gfp. **P < 0.01 versus AAV5-Rmst + Scr group.

To further confirm whether *Rmst* is responsible for stabilizing *Dnmt3a* mRNA in the damaged DRG, we inhibited Dnmt3a expression through delivering Dnmt3a siRNA into the DRG after AAV5-Rmst microinjection. We found blocking *Rmst* overexpression-induced Dnmt3a increase could not lower *Rmst* expression (Figure 6A) [*F*(2,6) = 19.28] but lead to a fall of *Dnmt3a* mRNA and protein (Figures 6B,C) [Figure 6B: *F*(2,6) = 62.27; Figure 6C: *F*(2,6) = 31.06]. More importantly, blocking Dnmt3a expression attenuated the *Rmst* induced-nociceptive hypersensitivity (Figures 6D–F) [Figure 6D: *F*(8,60) = 13.12; Figure 6E: *F*(8,60) = 3.500; Figure 6F: *F*(8,59) = 10.35]. Collectively, nerve injury induced *Rmst* upregulation participates in NP by stabilizing the *Dnmt3a* mRNA expression.

### Rhabdomyosarcoma 2-associated transcript regulates the DNA methyltransferase 3 alpha mRNA stability by interaction with HuR under neuropathic pain condition

Finally, we asked the potential mechanism of *Rmst* induced Dnmt3a upregulation in injured DRG neuron. Given that RNA-binding proteins (RBPs) affect the targeted mRNA stability, we focused on one of well-characterized RBPs, HuR. Notably, HuR was reported as a contributor to nociceptive pain (Kunder et al., 2022) and an anti-HuR could alleviate nerve-injury induced NP (Borgonetti and Galeotti, 2021). In particular, it was HuR reported to stabilize the *Dnmt3* mRNA (Peng et al., 2020). Therefore, under NP condition, we asked whether HuR contributed to *Rmst*-mediated increased Dnmt3a.

**FIGURE 7:**
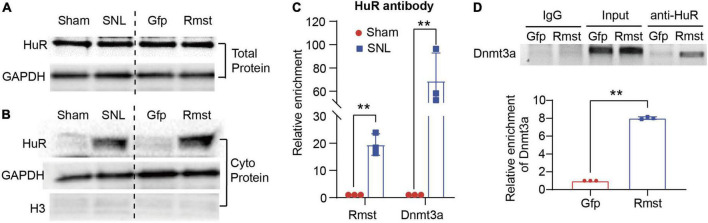
Rhabdomyosarcoma 2-associated transcript (*Rmst*) was interaction with HuR to regulate the *Dnmt3a* mRNA Stability Under NP Condition. **(A,B)** Level of HuR expression in total protein **(A)** and cytoplasm protein **(B)** in DRG with SNL model and AAV5-Rmst (AAV5-Gfp as control) DRG microinjection. **(C)** Level of *Rmst* or *Dnmt3a* mRNA immunoprecipitated by anti-HuR on day 7 post-SNL surgery *n* = 3 biological repeats. **P < 0.01 versus sham group. **(D)** Level of *Dnmt3a* immunoprecipitated by HuR in DRG neuron with AAV5-Rmst. *n* = 3 biological repeats. **P < 0.01 compared with AAV5-Gfp group.

We first determined the alteration of HuR in DRGs after SNL or *Rmst* overexpression. Unexpectedly, we found neither SNL nor *Rmst* could regulate the HuR expression in injured DRG (Figure 7A). In fact, in many cancerous settings, HuR was increased subcellular localization within the cytoplasm to stabilize various prosurvival mRNA (Schultz et al., 2020). Therefore, we examine whether SNL induced cytoplasmic accumulation of HuR. We found SNL caused a steep increase of HuR protein in the cytoplasm of the damaged DRG while *Rmst* overexpression in DRG was found similar phenomenon (Figure 7B). This may suggest that under NP condition, HuR is transported from the nucleus to cytoplasm. Next, RNA immunoprecipitation (RIP) assay revealed that HuR was capable to enrich *Rmst* and *Dnmt3a* in SNL group, approximately 20-fold, and 60-fold, respectively, compared to sham group (Figure 7C). Finally, we found overexpression of *Rmst* in primary neuron promoted the binding between *Dnmt3a* mRNA and HuR (Figure 7D). Thus, *Rmst* appeared to be the essential regulator that promoted Dnmt3a expression through interacting with HuR in DRG neuron.

## Discussion

**FIGURE 8:**
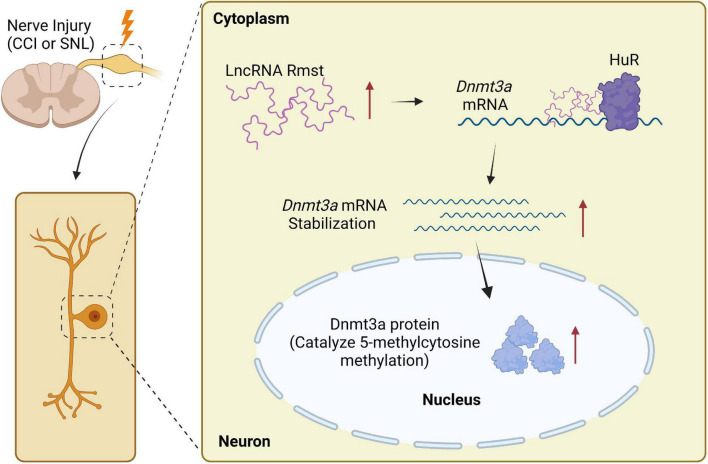
Proposed mechanism on rhabdomyosarcoma 2-associated transcript (*Rmst*) in NP. Nerve injury-triggered *Rmst* upregulation interacts with HuR in injured DRGs, resulting in stabilizing *Dnmt3a* mRNA and protein. The latter one has been reported as a critical NP regulator in DRG neurons. In contrast, in normal DRG neuron, *Dnmt3a* mRNA would be degraded without *Rmst*.

This is the first study to examine the molecular and cellular function of *Rmst*, a lncRNA in injured DRG neuron that modulates NP. Specifically, the increased *Rmst* was positively regulated Dnmt3a by promoting its mRNA stability and interacting with HuR, which leads to NP. Blocking the elevation of *Rmst* could reverse nerve injury-induced Dnmt3a upregulation and alleviate pain hypersensitivities (Figure 8). The present study suggests that *Rmst* in DRGs is likely a key regulator in NP.

In 2013, the first lncRNA involved in NP was reported in detail (Zhao et al., 2013, p. 2). After that, the regulation of lncRNA on NP are popping up over the past few years (Wu et al., 2019). And we are the first to report lncRNA *Rmst* is involved in the NP. In fact, *Rmst* was first identified as non-coding RNA in 2012, and it was essential for neuronal specification in human embryonic stem cells (Ng et al., 2012). Under normal condition, *RMST* in both human and mouse occurs primarily in CNS (Uhde et al., 2010; Ng et al., 2012) and, more importantly, is abundant in neuron (Julian et al., 2013, p. 2; Briese et al., 2016). *RMST* physically interacts with SOX2 and regulates neural fate by regulating neurogenesis related genes (Ng et al., 2013, p. 2). *RMST* deficiency in neural stem cells resulted in glia differentiation (Ng et al., 2013), indicating that *RMST* is important for neural differentiation in particularly during brain development period. However, increased *Rmst* expression was reported in CNS diseases, including stroke (Zhao et al., 2021; Li et al., 2022) and Parkinson’s disease (Ma et al., 2021). Blocking *Rmst* expression could protect from neuronal apoptosis and improve neurological function (Ma et al., 2021; Zhao et al., 2021; Li et al., 2022), which suggests that excessive *Rmst* expression may cause CNS disease aggravations. Our present study found *Rmst* was also increased expressed in DRG neuron under NP condition. More importantly, blocking *Rmst* expression in injured DRGs could mitigate nerve injury-induced nociceptive hypersensitivity. However, why Rmst siRNA in DRGs did not alter response to mechanical and heat stimuli is unclear, which may be due to low *Rmst* expression in physiological condition. Together, the strong evidence indicates that the dysregulation of *Rmst* in neuron may contribute to NP development.

Evidence has been emerged lncRNAs mediates DNA methylation in various pathological condition (Huang et al., 2022), including schizophrenia (Ni et al., 2021, p. 006), diabetic retinopathy (He et al., 2021, p. 3), colon cancer (Merry et al., 2015) and so on. In particular, *RMST* was characterized as a positive regulator for DNMT3 but not DNMT1 by increasing *DNMT3* mRNA stability in cancer (Peng et al., 2020). In our study, *Rmst* upregulation may contribute to the nerve damage-triggered Dnmt3a increase by stabilizing its mRNA in NP. Notably, when *Rmst* was overexpressed in DRG neuron, only DNMT3a but not DNMT3b appear to generate, and blocking DRG *Rmst* expression abolished SNL-induced DNMT3a upregulation, which was consistent with previous study (Peng et al., 2020, p. 3). Furthermore, *Rmst* did not directly modulate nociceptive hypersensitivity as in absence of Dnmt3a expression in DRG neuron the overexpression of *Rmst* failed to completely mimic nerve damage-induced nociceptive hypersensitivity. In fact, it has been reported DNMT3a in DRG neurons is involved in the NP (Guo et al., 2019). Knockout DNMT3a in DRG significantly attenuated nociceptive hypersensitivity (Zhao et al., 2017). What’s more, in NP, transcriptional factors, such as CREB (Yang et al., 2021) and Oct1 (Zhao et al., 2017), could bind to the promoter region of Dnmt3a to boost Dnmt3a expression. Our study demonstrated that *Rmst* is required for the stability of *Dnmt3a* mRNA, enhancing DNMT3a expression. It should also be noted that *Rmst* also regulates other NP related genes including sex-determining region Y-box2 (*Sox2*) (Ng et al., 2013, p. 2; Zhang et al., 2019, p. 2) and heterogeneous nuclear ribonucleoprotein D (*hnRNPD*) (Liu et al., 2020; Feng et al., 2021). Whether these genes are also regulated by *Rmst* in NP remains to be determined.

Mechanistically, nerve injury-induced elevated *Rmst* could recruit HuR protein, thereby stabilizing the *Dnmt3a* mRNA and reducing the *Dnmt3a* mRNA degradation. HuR, as an RNA-binding protein, is capable to stabilize AU-rich elements (AREs)-containing reporter mRNA in the cytoplasm through binding AREs sequences (Gallouzi et al., 2000). The new finding has pointed out anti-HuR delivery has been proven effective to relieve pain hypersensitivity by inhibiting spinal neuroinflammation (Borgonetti and Galeotti, 2021). Therefore, the effectiveness of analgesics of anti-HuR may also be due to the degradation of *Dnmt3a* mRNA. However, further experiments are needed.

In conclusion, our study indicated that blocking *Rmst* expression in injured DRGs mitigated NP at least in part through enhancing degradation of *Dnmt3a* mRNA. Thus, *Rmst* may become a promising target and provide insightful directions for NP treatment.

## Data availability statement

The RNA sequencing dataset for mouse DRG after peripheral nerve injury was obtained from previous research (https://doi.org/10.1177/1744806916629048). The ScRNA-seq dataset for mouse DRG after peripheral nerve injury was from Gene Expression Omnibus (GEO) with the series record GSE155622.

## Ethics statement

This animal study was reviewed and approved by Guangzhou Medical University.

## Author contributions

XG and XS conceptualized and designed the study. XG and WC contributed to write the manuscript. GZ and FH performed animal model and behavior test. XG and JQ performed molecular experiments. All authors read and approved the final manuscript.
