# Supplementary material for: Long non-coding RNA rhabdomyosarcoma 2-associated transcript contributes to neuropathic pain by recruiting HuR to stabilize DNA methyltransferase 3 alpha mRNA expression in dorsal root ganglion neuron
Source: Front Mol Neurosci. 2023 Feb 22;15:1027063. doi: 10.3389/fnmol.2022.1027063 (PMC9992530; doi:10.3389/fnmol.2022.1027063)
Supplement: Supplementary file 1 [file Table_1.DOCX]

Supplementary Table1

| Rmst-F’ | TGGCATCTATCCGGTGCTTC |
| --- | --- |
| Rmst-R’ | CAACCAATTGCAGCCGACTC |
| Dnmt3a-F’ | GAGGGAACTGAGACCCCAC |
| Dnmt3a-R’ | CTGGAAGGTGAGTCTTGGCA |
| Dnmt3b-F’ | AGCGGGTATGAGGAGTGCAT |
| Dnmt3b-R’ | GGGAGCATCCTTCGTGTCTG |
| Gapdh-F’ | AGGTCGGTGTGAACGGATTTG |
| Gapdh-R’ | TGTAGACCATGTAGTTGAGGTCA |
| Tuba1a-F’ | GTGCATCTCCATCCATGTTG |
| Tuba1a-R’ | GTGGGTTCCAGGTCTACGAA |
| Malat-F’ | TGAAAAAGGAAATGAGGAGAAAAG |
| Malat-R’ | CTTCACAAAACCTCCCTTTACAAT |
| siRmst-SE | CAGUUAGUAAAUCAACGAATT |
| siRmst-AS | UUCGUUGAUUUACUAACUGTT |
| siDnmt3a-SE | UCUUGAGUCUAACCCCGUGTT |
| siDnmt3a-AS | CACGGGGUUAGACUCAAGATT |
