# Supplementary figures and images for: Long non-coding RNA rhabdomyosarcoma 2-associated transcript contributes to neuropathic pain by recruiting HuR to stabilize DNA methyltransferase 3 alpha mRNA expression in dorsal root ganglion neuron
Source: Front Mol Neurosci. 2023 Feb 22;15:1027063. doi: 10.3389/fnmol.2022.1027063 (PMC9992530; doi:10.3389/fnmol.2022.1027063)

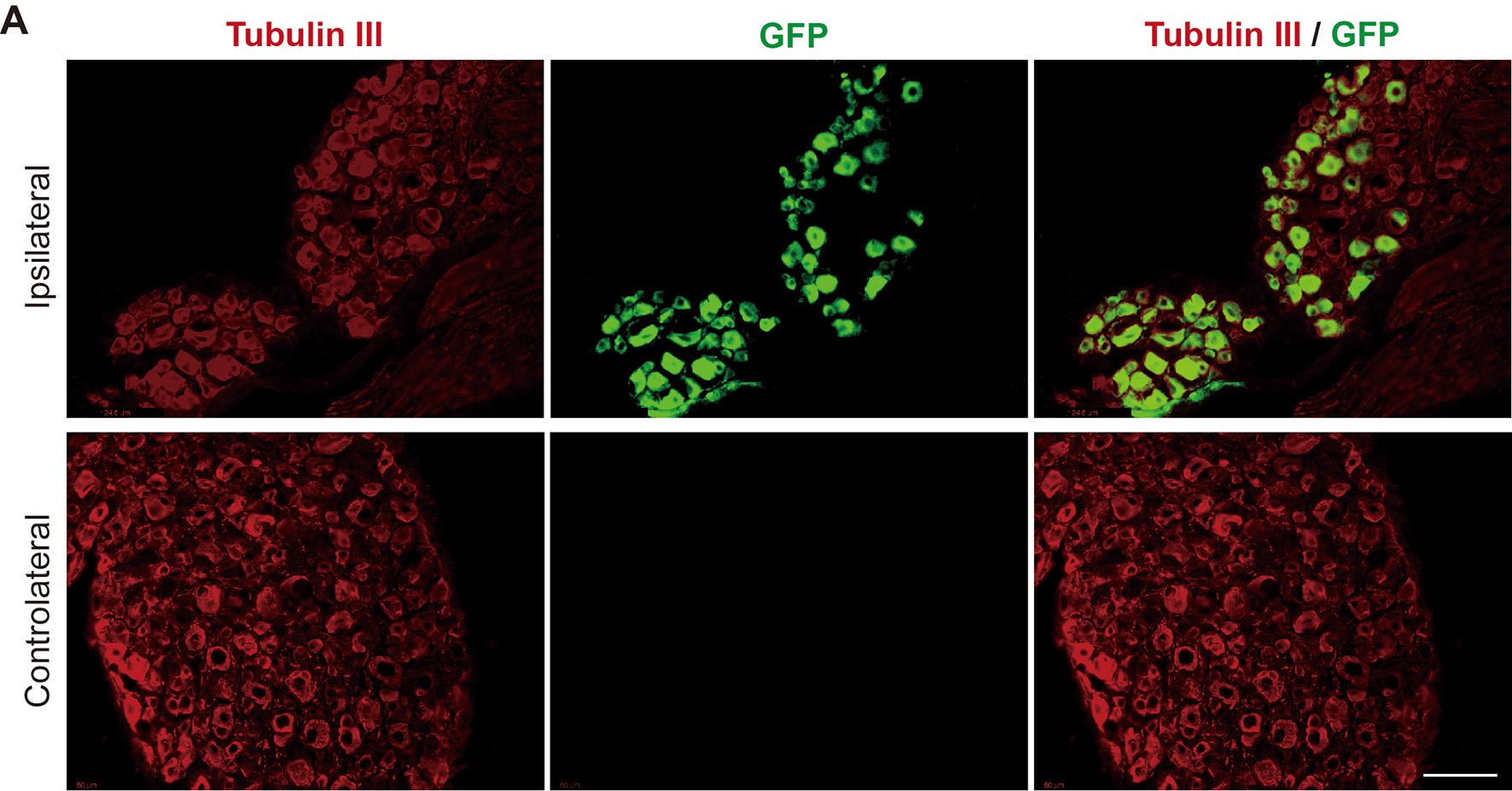

Supplement: Supplementary file 2 [file Image_1.JPEG]
